# 

**Published:** 1897-10

**Authors:** 


### Society Notes.

---

**Do not** forget that the National Prison Association will hold its 21st annual session in Austin, on 16th to 20th of present month, October. A large attendance is expected, and the meeting will be a most important one. The subject of Criminal Law Reform being the one which will receive most considera tion. An invitation is extended to the doctors to attend.

**American Academy Railway Surgeons.**—The 24th Annual meeting of this Association will be held in the auditorium at Chicago, October 6th, 7th and 8th. President, L. E. Lemen. M. D., Denver; Secretary, D. C. Bryant, M. D., Omaha.

---

### Tri-State Medical Society.

Dear Doctor:—You are invited with your friends to attend the Ninth Annual meeting of the Tri-State Medical Society of Alabama, Georgia and Tennessee, to be held in the Auditorium of the Tulane Hotel, corner Church and Spruce streets, Nashville, Tenn., Tuesday, Wednesday and Thursday, October 12, 13, and 14, 1897.

If you desire to read a paper, report a case or exhibit a specimen, notify the secretary.

Reduced railroad rates to the Tennessee Centennial.

Frank Trester Smith, M. D., Secretary, Chattanooga, Tenn.

W. F. Westmorland, M. D., President, Atlanta, Ga.

W. D. Haggard, Jr., M. D., Chairman Committee of Arrangements, Nashville. Tenn.

---

### Brazos Valley Medical Association.

Easterly, Robertson Co., Texas, Sept. 20, 1807.

Brazos Valley Medical Association, fourth annual session, convenes at Navasota, Texas, the second Tuesday and Wednesday in November next, same being the ninth and tenth of the month:

Dear Doctob:—We are flattered with the prospect of a pleasant and profitable meeting. Lay aside the cares of life for two days and join with us in making this one of the most successful and interesting sessions ever held by our association. We need your assistance and cordially invite you to be present. The physicians of Navasota are exerting themselves to entertain every one who may attend. The meeting will be called to order promptly at 10 o'clock, a. m. Now, Doctor, don't fail to come and bring your wife with you. For any desired information apply to Dr. G. M. Abney, president, B. V. M. A., Franklin, Texas; Dr. D. L. Peeples, vice-president, B. V. M. A., Navasota, Texas, or to Dr. W. B. Briggs, secretary, B. V. M. A., Easterly, Texas.

TO OUR SUBSCRIBERS.—With this issue we send out every bill on our books, and we will draw on a good many delinquents. We earnestly call on our friends and patrons to settle their arrears at least. We have indulged them all through the dead season of summer, and for three years have not pressed any one for payment; hence many are in arrears who usually pay in advance, and the JOURNAL feels the need of the money. We must *earnestly insist on payment*, in part, at least, and if for no other reason—to show appreciation of our indulgence. But *we need the money*. We have given you a good, lively, up-to-date JOURNAL—paying cash with the delivery of each edition, and it takes money to do that. Pay or no pay, the JOURNAL, like Tennyson's brook, has gone on and goes on, forever. "The cold wind doth blow"—(that is, it is *going* to blow now "pretty quick, right away") and "we shall have snow (may be, byme-bye, p'raps) and what poor Robin is going to do then (unless our subscribers come to the rescue), is one of the unsolved problems that is keeping us awake nights. Send along your subscriptions for back rations if you can't pay ahead like you used to do. F. E. D.

